# Preventing a global health care systems collapse through low-tech medicine

**DOI:** 10.7189/jogh.14.03035

**Published:** 2024-08-09

**Authors:** Marine Sarfati, Anne Senequier, Philippe Bihouix, Laurie Marrauld, Romain Manet

**Affiliations:** 1 Department of Public Health, University Hospital of Lyon (Hospices Civils de Lyon), France; 2 Optional Teaching of Environmental Medicine and Health, University of Lyon, France; 3 Observatory on Health and Climate, French Institute for International and Strategic Affairs, Paris, France; 4 Private practice, Paris, France; 5 AREP Group, Post Carbon architecture, engineering, consulting, Paris, France; 6 Independent researcher, Bordeaux, France; 7 EHESP – French School of Public Health, Rennes, France; 8 Ministry of Health and High Council for the future of Social Security, Paris, France; 9 Department of Cranial Neurosurgery, Neurological Hospital Pierre Wertheimer, University Hospital of Lyon (Hospices Civils de Lyon), France; 10 Department of Neurosurgery, Military Teaching Hospital Sainte-Anne, Toulon, France

## PAST

Human life expectancy, which had been stagnant at around 30–40 years for approximately 300 000 years, surged over the past two centuries. Today, it averages 73 years worldwide and exceeds 80 in countries within the Organisation for Economic Co-operation and Development (OECD). The remarkable progress in health has been driven by interconnected factors dependent on concerted efforts from the medical, public, and economic sectors. This has manifested in socioeconomic advancements (improved living standards, education, and economic conditions), public health initiatives (implementation of sanitation measures; food and water safety regulations; and the establishment of organised healthcare systems, health insurance, and institutions like the World Health Organization (WHO)), and medical innovations (developments in pharmaceuticals, especially vaccines and antibiotics; technological advancements such as X-rays, MRI, and surgical devices; and enhancements in the education of healthcare professionals and medical research, facilitating the rapid dissemination and adoption of new medical practices).

Overall, these factors have largely been enabled by the rapid economic expansion following the Industrial Revolution, which in turn resulted from a fast increase in energy use [1], particularly fossil fuels, as well as other non-renewable resources.

## PRESENT

In 1972, Meadows et al. [2] demonstrated through computerised modelling that endless economic expansion in a resource-limited world was unsustainable, foreseeing a potential collapse by the mid-21st century. Until today, their model has accurately predicted the trajectory of societies. The crossing of the ‘peak oil’ in 2020 for nearly all producing regions [3], in addition with mandatory energy transition to mitigate climate change, marked a scarcity of easily accessible, affordable energy. The war in Ukraine acutely highlighted the consequences of such shortages. This structural decrease in ‘cheap energy’ will have growing irremediable impacts on global economies, consequently affecting health care systems. For instance, the decline in conventional crude oil (cheap energy) production since 2005 [4] has been a major driver of the 2007 ‘subprime crisis’ in the USA and the ensuing global financial crisis in 2008 [5]. The repercussions of this crisis continue to impact economies worldwide, leading governments to reduce public spending in order to shrink budget deficits, including through cuts to funding for health and social care [6]. In European countries, which are heavily reliant on oil imports, these constraints are exemplified by the uninterrupted reduction in the number of hospital beds, as observed in the UK [7] and France [8] since the late 2000s.

Additionally, there are growing disparities between resources consumption and health progress. For instance, between 2000 and 2019 health spending globally rose over 223%, while life expectancy increased just 9.9% [9].

Over the past two centuries, in parallel with industrial and economic growth and bolstered by increased wealth, comfort, and security, Western societies have experienced significant changes in the perception and acceptance of risk [10] and death [11], and a simultaneous evolution of medicine [12]. The consumption of health care in these developed countries has continuously risen, and not solely due to an ageing population. These changes are reflected in many aspects, such as the legal frameworks and the growing impact of medico-legal issues on health care management. Although the direct costs of the medical liability system represent a small fraction of overall health expenditures, the broader implications – such as the practice of ‘defensive medicine’ – likely have a more significant impact on the cost and quality of care [13].

Furthermore, medicine has become a very profitable business, especially as the focus of medical innovation has shifted from solving health issues to prioritising financial gains. Many health facilities are now owned by private hedge funds, driven by short-term profit goals rather than sustained, strategic investments. The medtech and biotech industries are often driven by the allure of fashionable, expandable high-tech breakthroughs lacking proven value. Robotic surgery is an emblematic example [14]. These ‘advancements’ then become the focus of marketing competition among health care providers, promoted through polished stories on social networks. In this context, the absence of nationwide oversight of investments leads to paradoxical scenarios where some regions have redundant medical resources, while lacking essential supplies. Additionally, intermediaries (such as insurers, chemists, drug distributors, and pharmacy-benefit managers) are capturing an increasingly larger share of the overall health costs, with the part of their revenue in the total health bill in the US rising from 25% in 2013 to 45% in 2022 [15]. From a long-term perspective, the commodification of health and medical care has proven to be a failure [16]. It has also largely contributed to destroying the primary health care providers’ motivation. The loss of meaning, combined with the unsustainable conditions (often mislabeled as 'optimisation') of their work, has certainly contributed to the dramatic and growing shortage of health care professionals worldwide. In addition, unlike other sectors, health care has undergone minimal automation. Consequently, this strain on the health care workforce is particularly troubling.

Finally, a range of ‘invisible’ socioeconomic and environmental factors across various sectors – including energy, education, water, sanitation, urban planning, agroindustry, and marketing – have increasingly become detrimental to health without being directly recognised as such.

## FUTURE

Moving forward, the sustainability of our current level of health care is not guaranteed – not even in high-income countries. This is primarily due to two reasons. First, as we previously mentioned, increasing shortages of affordable energy are already impacting the funding of health care systems, including those in OECD countries, leading to a contraction in both material and human resources. Second, health systems in these countries are significant contributors to unsustainable environmental impacts, which have continuously increased in recent years [17]. The health sector accounts for nearly 5% of global greenhouse gas emissions [18]; this figure rises to 8–10% of national emissions in more developed countries [19,20].

Early warning signs, such as the rising infant mortality rate in a high-income country like France [21], the resurgence of ‘Victorian-era diseases’ related to poor socio-economic conditions such as scurvy, rickets, scabies and measles in the UK [22], and decreased life expectancy in the US [23], could signal the beginning of a broader decline. Similar to climate change, the great inertia of these complex systems delays the visible impact of ongoing disruptive mechanisms.

Immense challenges also threaten human health and health care systems worldwide, including natural disasters linked to climate change, restrictions on water and food supplies, pandemics, wars, and pollution. The lattermost is the main environmental cause of disease and premature death in the world today, responsible for an estimated nine million premature deaths in 2015, representing 16% of all deaths worldwide [24].

Health needs will become less predictable and more volatile over time, necessitating significant adaptability from health care providers. In their current organisation, our exhausted health systems will not be able to cope with these issues. To prevent the complete collapse of health care systems and adapt them to tomorrow’s challenges, we need to develop a proactive, cross-disciplinary approach which will surpass the outdated paradigm of perpetual growth and the distorted notion of progress, aiming to durably maintain the current level of care – a feat which would in itself be a significant achievement.

This approach will continue to be supported by selected high-tech solutions, provided they prioritise genuine progress over profit or competition. More critically, there is an urgent need to conceive and develop strategies that could be grouped under the concept of ‘low-tech medicine’. This approach should be based on a transdisciplinary methodology that integrates both medical and non-medical expertise, such as public health, engineering, sociology, education, ethics, urban planning, etc.

Our concept of low-tech medicine intersects with several emerging concepts. These include, for example, Realistic Medicine [25] and related initiatives such as Prudent Healthcare [26], Slow Medicine [27], Choosing Wisely [28] and Less is More [29], which emphasise shared decision-making, care personalized to local contexts, a shift from ‘diagnosis-centred care’ to ‘best management strategy’, and the training of local health care professionals. It also encompasses environmental health, which focuses on the reciprocal impacts between the environment and health care.

‘Low-tech medicine’ further expands upon these ideas by developing four strategic axes:

− Redefining care by allowing healthcare providers to deliver practical, accessible, resilient, and sustainable care, while also educating the population to value healthcare as an essential service rather than a commodified good. In this framework, our relationship to disease, pain, death, and risk will have to be reconsidered from ethical perspectives;
− Reorganising the healthcare ecosystem to optimise resources, de-implement low-value care, minimise environmental impact, and address the invisible health issues of the Anthropocene. Preventive medicine, including societal intervention targeting sedentary lifestyle, diet, physical activity, and psychological well-being, is typically a very efficient low-tech approach and should be much more developed;
− Promoting low-tech research and development at both academic and industrial levels, while simultaneously advancing high-tech research that genuinely drives progress;
− Implementing strategic foresight to anticipate future health needs and resources and to forge a new narrative that moves beyond individual-centric models to a cohesive societal framework where health is reconsidered as a pillar, not merely an adjustment of financial variables.

Within this approach – which could be developed in both high-income and low-income countries – the term ‘low-tech’ corresponds not only to ‘low-technology’, but also to ‘low-techniques’, ‘low-technicity’, and ‘low-technicality’, and even to ‘low-technocracy’, in the sense of simplifying and optimising health care organisation. In this regard, the response to coronavirus disease 2019 (COVID-19) pandemic serves as an excellent source of inspiration. It demonstrated the effectiveness of a simplified, coordinated collective response characterised by decentralized decision-making (‘low-technocracy’) and the empowerment of non-expert individuals – the general population (‘low-technicity’). Key strategies employed were predominantly ‘low-technology’ solutions such as masks and hand-washing, coupled with basic techniques (‘low-techniques’) like patient isolation and oxygen delivery.

Similar to other fields explored through the low-tech lens [30], our concept of ‘low-tech medicine’ embodies accessible, sustainable, efficient, and sober care. It addresses key sustainability challenges in health care by optimising care cost-effectively and minimising environmental impacts. Ultimately, its inherent resilience allows it to adapt more effectively to unpredictable health challenges. Therefore, this strategy closely aligns with the WHO's promotion of climate-resilient health systems ‘as those capable of anticipating, responding to, coping with, recovering from, and adapting to climate-related shocks and stress, while minimizing GHG [greenhouse gas] emissions and other negative environmental impacts to deliver quality care and protect the health and well-being of present and future generations’ [31].

However, this approach can be divisive among caregivers and the general population due to its disruptive nature, especially in OECD countries. Physicians, trained to deliver uncompromised care, may resist adopting what seems like a ‘downgrade’, while patients accustomed to prompt solutions might reject ‘less advanced care’. This leaves an essential need to inform health care providers about the current and future challenges, as well as the unsustainability of modern health care. Similar to financial debt, refusing to slightly simplify care today will necessitate much more significant downgrades tomorrow. Additionally, extensive educational efforts should promote societal accountability and a realistic relationship with disease and death among the general population. This would encourage collaborative efforts with industrial actors and policymakers to revalue essential yet ‘less profitable’ care.

There is an urgent need to reset our mindset, heeding the words of Gaston Berger, the French philosopher and pioneer of prospective (foresight): ‘Tomorrow will not be like yesterday. It will be new and depend on us. It is less to be discovered than to be invented.’
